# Supplementary figures and images for: Non-invasive monitoring of glucocorticoid metabolite concentrations in urine and faeces of the Sungazer (Smaug giganteus)
Source: PeerJ. 2018 Dec 21;6:e6132. doi: 10.7717/peerj.6132 (PMC6305116; doi:10.7717/peerj.6132)

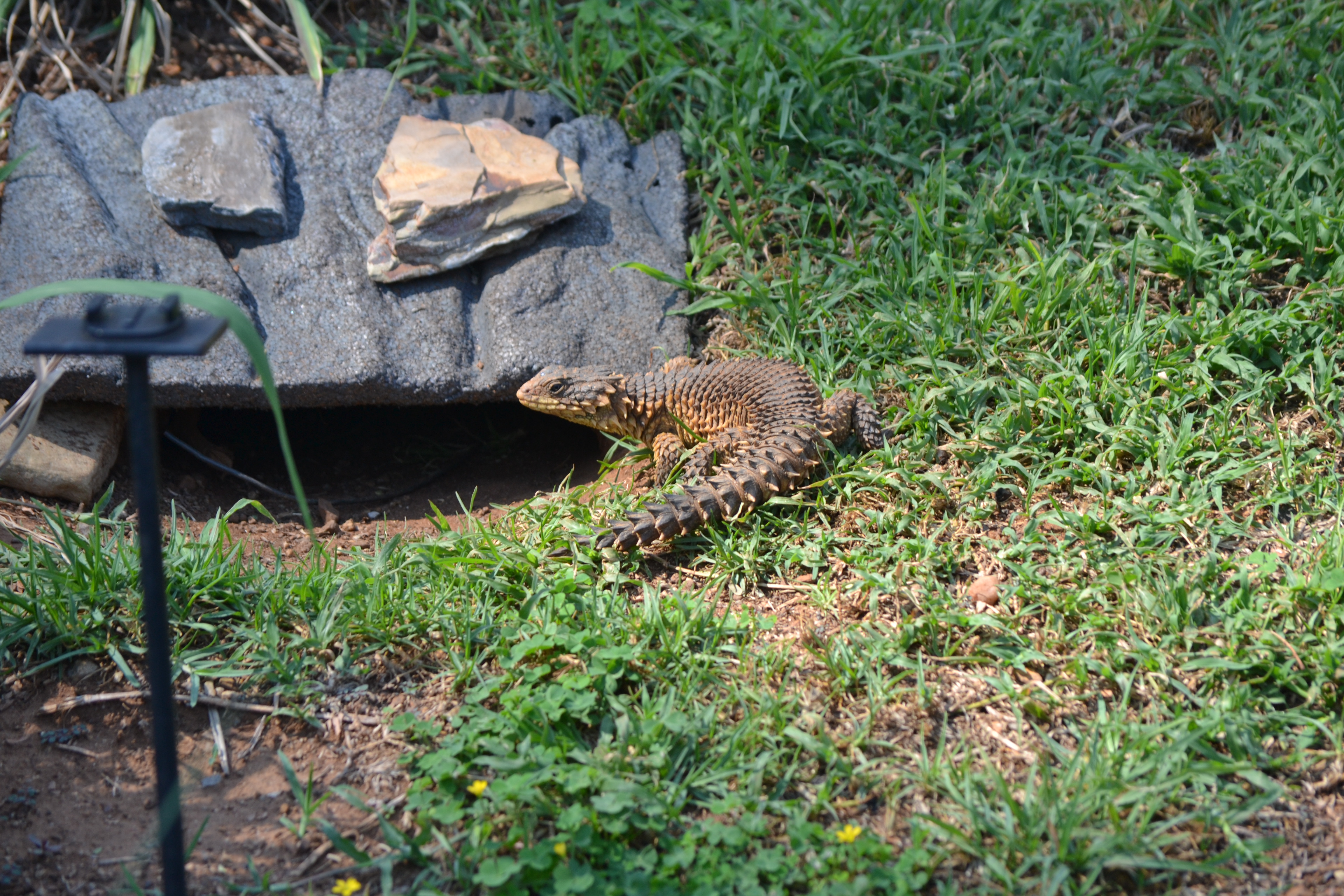

Supplement: Figure S1 [file peerj-06-6132-s001.jpg]

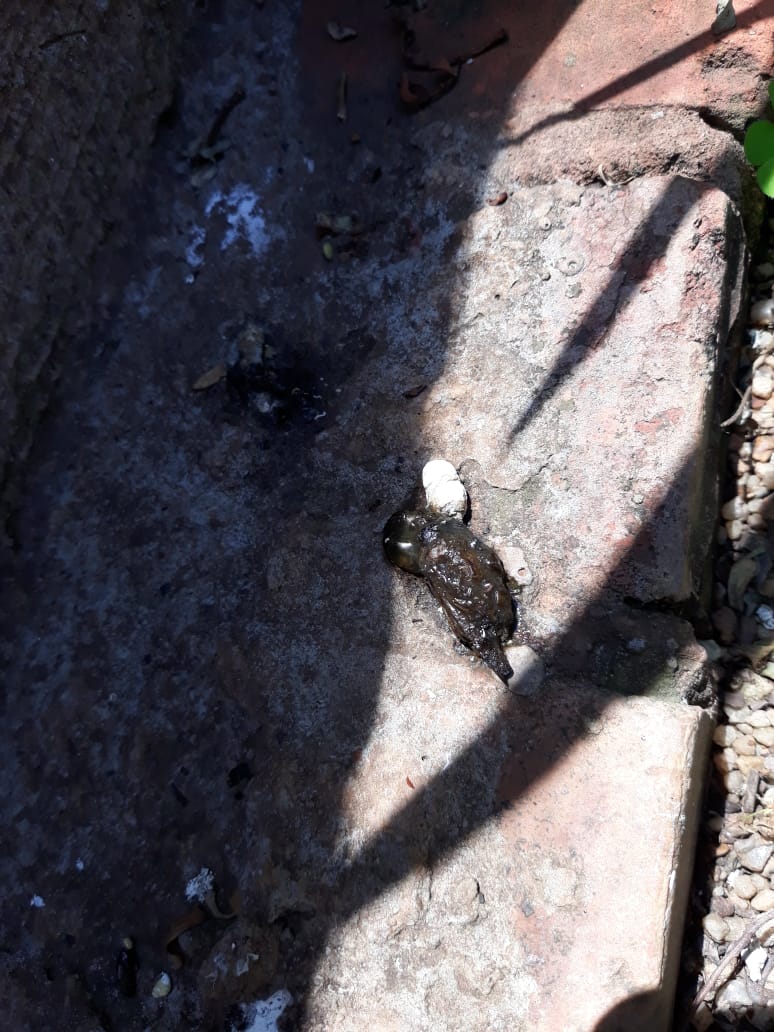

Supplement: Figure S1 — The white component represents urine, while the dark section represents faeces (Photographer: Juan Scheun) [file peerj-06-6132-s002.jpg]
